# Emergency Dyspnea in 258 Cats: Insights from the French RAPID CAT Study

**DOI:** 10.3390/vetsci12030242

**Published:** 2025-03-03

**Authors:** Nour Abboud, Jack-Yves Deschamps, Marie Joubert, Françoise A. Roux

**Affiliations:** 1 Emergency and Critical Care Unit, Oniris VetAgro Bio, Nantes-Atlantic College of Veterinary Medicine, Food Science and Engineering, La Chantrerie, CEDEX 03, 44307 Nantes, France; nour.abboud@oniris-nantes.fr (N.A.); marie.joubert@oniris-nantes.fr (M.J.); francoise.roux@oniris-nantes.fr (F.A.R.); 2 NP3, Nutrition, PathoPhysiology and Pharmacology Unit, Oniris VetAgro Bio, Nantes-Atlantic College of Veterinary Medicine, Food Science and Engineering, La Chantrerie, CEDEX 03, 44307 Nantes, France

**Keywords:** feline dyspnea, pleural effusion, feline cardiology, cat, emergency, critical care

## Abstract

Dyspnea is common in feline emergency medicine. Dyspneic cats are often unable to tolerate additional diagnostic tests upon admission. The aim of this study was to determine the relative frequency of the causes of severe dyspnea in cats and to identify clinical indicators that could guide immediate management. Records of 312 severely dyspneic cats admitted over a 5-year period to our institution’s emergency and critical care unit were retrospectively reviewed. The cause of dyspnea was identified for 258 cats, representing 83% of the cases. This study focused solely on these 258 cats. Respiratory causes accounted for 33% of cases, followed by cardiac causes (25%) and both traumatic and neoplastic causes (21% each). Pleural effusion was present in 39% of the cats, with various origins: cardiac (38%), respiratory (pyothorax, feline infectious peritonitis, 30%), neoplastic (23%), and traumatic (9%). Male cats were significantly overrepresented in the cardiac and respiratory groups. The median age differed according to the cause (2 years for traumatic causes, 6 years for respiratory causes, and approximately 11 years for cardiac and neoplastic causes). Cats with cardiac disease were significantly more often hypothermic. Hyperthermia was not systematically indicative of an infectious process. No clinical element (temperature, heart rate, cardiac auscultation, etc.) allowed for predicting the cause of dyspnea. In-hospital mortality was 44%, varying according to the cause (78% for neoplastic causes, 40% for traumatic causes, 37% for cardiac causes, and 30% for respiratory causes). In conclusion, the four main categories of causes were represented in approximately equal proportions, which contrasts with the results of the British RAPID CAT study, where cardiac causes accounted for nearly two-thirds of dyspnea cases. In the dyspneic cats in this series, the clinical presentation did not allow clinicians to identify a particular cause. This highlights the importance of non-invasive examinations such as POCUS (point-of-care ultrasound), which has become a routine test in emergency medicine. However, this importance should not lead to underestimating the continued value of radiography when the cat’s clinical condition allows for image acquisition.

## Simple Summary

A retrospective 5-year analysis of 258 dyspneic cats with an etiological diagnosis identified four main causes: respiratory (33%), cardiac (25%), traumatic (21%), and neoplastic (21%). Pleural effusion was common (39%) with varied origins. The main difference between the groups was the mean age: 2 years for traumatic causes, 6 years for respiratory causes, and approximately 11 years for cardiac and neoplastic causes. No clinical indicator reliably predicted the cause of dyspnea. The in-hospital mortality rate was 44%, varying according to the cause. The results of this study highlight the limitations of interpreting clinical signs in severely dyspneic cats and emphasize the importance of non-invasive examinations such as POCUS (point-of-care ultrasound) and the continued relevance of radiography.

## 1. Introduction

Severe dyspnea is defined as respiratory difficulty characterized by abnormalities in the frequency, amplitude, or rhythm of breathing movements. Dyspnea is a common clinical presentation in emergency veterinary medicine for cats. This study focuses exclusively on severe dyspnea, meaning cases likely to lead, within a short time, to respiratory arrest, cardiac arrest, and ultimately death. Dyspnea is the clinical manifestation of impaired gas exchange, reflecting a respiratory disorder in which the lungs fail to properly exchange oxygen and carbon dioxide. For optimal management, understanding the mechanism of dyspnea—and ideally its cause—is essential. However, the critical condition of dyspneic cats often makes complementary tests, such as radiography, ultrasound, or blood sampling, impractical; even minor investigations can precipitate decompensation and result in death. Most severely dyspneic cats examined in emergency settings have no prior history of respiratory difficulties to guide treatment.

Based on the underlying cause, dyspnea can be categorized as having a (1) cardiac, (2) respiratory, (3) neoplastic, or (4) traumatic origin. Knowledge of the relative frequency of these causes, as well as the specific clinical presentations associated with each, would allow clinicians to anticipate the origin of the dyspnea and initiate probabilistic treatment while awaiting complementary diagnostic testing.

Only two studies [1,2], both from the United Kingdom, have examined the relative frequency of different causes of dyspnea in cats. The first study, published in 2009, conducted by Swift et al. [1] in Liverpool (England), retrospectively reviewed the causes of dyspnea in 90 dyspneic cats referred to a specialty center over four years (2003–2007). The study identified the causes as 38% cardiac diseases (*n* = 34), 32% respiratory conditions (*n* = 29), 20% neoplasms (*n* = 18), and 9% trauma (*n* = 8). Only 58% of the cats were admitted for emergency care. The fact that these were referral cases could constitute a recruitment bias, as less severe cases may have been treated by primary veterinarians, while the most critical cases may have been euthanized earlier. The second study, published in 2018, conducted by Dickson et al. [2] across nine primary care veterinary clinics in the United Kingdom, prospectively recruited 101 dyspneic cats over five years and five months (June 2011 to October 2016). A diagnosis was established in 92 cats, revealing 65% with cardiac diseases (*n* = 60/92), 16% with respiratory diseases (*n* = 15/92), 11% with neoplasms (*n* = 10/92), and 8% with trauma (*n* = 7/92). For nine cats (9%), an etiological diagnosis was not obtained. At the conclusion of this study, the authors proposed a diagnostic algorithm based on clinical data and cardiac POCUS.

No comparable study has been conducted in France. Intrigued by the high proportion of cardiac cases reported in the study by Dickson et al. [2] (65.21%), we aimed to examine the causes of severe dyspnea in cats admitted to the emergency service of our institution—a veterinary school located in a French metropolitan area with over 600,000 inhabitants. The first objective of this study was to retrospectively determine the relative frequency of causes of dyspnea in a population of severely dyspneic cats presented to an emergency veterinary service in a large French metropolitan area. The second objective of this study was to identify indicators (epidemiological data, history, clinical presentation) that could predict the origin and prognosis of severe dyspnea in cats, prior to performing confirmatory complementary examinations. These observations aimed to verify whether the diagnostic algorithm proposed by Dickson et al. [2] could be applied to our population of cats.

## 2. Material and Methods

### 2.1. Case Selection

All severely dyspneic cats presented to the emergency and critical care service of our institution (Oniris VetAgroBio, School of Veterinary Medicine, France) over a 5-year period, from 1 January 2018 to 31 December 2022, were retrospectively included in this study. Cats were considered severely dyspneic if, in the clinician’s subjective judgment, they were likely to die due to their dyspnea within 24 h. If the same animal presented multiple times with dyspnea, only the first episode was analyzed; in other words, only cases presenting with dyspnea for the first time were retained. Case selection was conducted using keywords referenced in the institution’s database, followed by a careful case-by-case review of consultation reasons.

Cats that did not receive oxygen therapy were excluded, as they were considered to not be severely dyspneic. Dyspneic cats admitted to other services (internal medicine, cardiology, imaging, surgery) who were not transferred to the emergency and critical care department for dyspnea management were also excluded.

Therapeutic management, results of biological analyses, hospitalization duration, prescribed treatments and medium- and long-term outcomes were not analyzed in this article.

### 2.2. Study Name

The study was named “The French RAPID CAT Study” for “Rapid Assessment in Physical examination In Dyspnoeic cats” in reference to the name given by Dickson et al. [2] to their British study.

### 2.3. Initial Management on Admission

Cats were admitted for consultation during both day and night, either referred or not, with a licensed veterinarian (intern or hospital assistant) and were subsequently reexamined by one of the senior clinicians in the institution—two professors of emergency and critical care (one board certified) and one resident of emergency and critical care.

**Figure 1:**
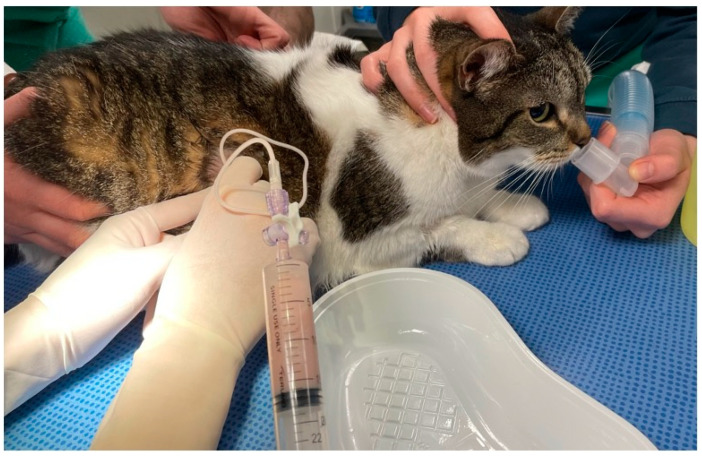
Thoracentesis of a pleural effusion, here a pyothorax, in an awake cat. In this series, 39% of the 258 dyspneic cats with an etiologic diagnosis had pleural effusion. Detecting pleural effusion is a priority, as thoracentesis provides significant relief.

The management followed a standard protocol for all dyspneic cats. It consisted of stabilization measures prior to determining the etiological diagnosis. Upon admission, all severely dyspneic cats received butorphanol intramuscularly (0.2–0.4 mg/kg) for sedation and were then placed at rest in a climate-controlled incubator for oxygen therapy. Once respiratory function improved, one or more rapid tests—thoracic POCUS, cardiac POCUS [3,4], and/or thoracic radiography—were performed, particularly to detect pleural effusion or left atrial dilation. If a significant volume of pleural effusion or a pneumothorax was observed, it was drained via thoracocentesis in conscious cats (Figure 1). More detailed radiographic, ultrasonographic, or cytological investigations were conducted only once the cat was stabilized, when possible. Biomarkers of cardiac distress, such as NT-proBNP and Troponin-I, were not used during the study period. No respiratory endoscopy was performed at presentation.

### 2.4. Classification of Dyspnea Causes

The origin of dyspnea was classified into five categories: cardiac (C), respiratory (R), traumatic (T), neoplastic (N), undiagnosed/unrelated (U). It is understood that, ultimately, all dyspnea results from pulmonary impairment; for instance, congestive heart failure causes dyspnea by provoking pulmonary edema, pleural effusion, or, more rarely, pulmonary artery thrombosis.

**Figure 2:**
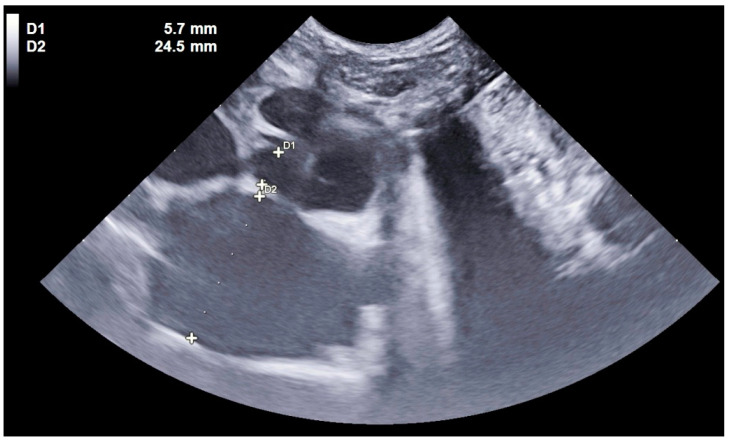
Atrial dilation confirmed by a left atrium-to-aorta ratio of 4.3 in a dyspneic cat. A cardiac POCUS, minimally stressful for the patient, allows for the detection of atrial dilation and thus confirms the cardiac origin of dyspnea.

Cats were classified into the cardiac group (C) if they presented with congestive heart failure for which echocardiographic diagnosis revealed cardiomyopathy (hypertrophic, restrictive, dilated, or unclassified), congenital heart disease, valvular disease, endocarditis, myocarditis, or pericardial effusion. Cats with cardiomegaly identified via cardiac POCUS by a left atrium-to-aorta ratio > 1.55 on a transaortic short-axis view [5] (Figure 2) or confirmed radiographically with a vertebral heart scale (VHS) score greater than 9.3 [6] were also included in this group. Cats with aortic thromboembolism without congestive heart failure were not included. Unlike the study by Dickson et al. [2], therapeutic response was not considered in diagnosing congestive heart failure.

Cats were classified into the respiratory group (R) if they had identified respiratory involvement not falling into the cardiac (C), traumatic (T), or neoplastic (N) categories. These included cats with non-cardiogenic, non-cancerous, and non-hemorrhagic pleural effusion (essentially pyothorax or feline infectious peritonitis), bronchopneumonia, pulmonary abscess, a thoracic foreign body, peritoneal–diaphragmatic hernia, non-cardiogenic pulmonary edema, or asthma. Asthma, as a functional disorder, was a diagnosis of exclusion based on the presence of severe dyspnea in the absence of cardiac abnormalities, pleural effusion, pneumothorax, thoracic masses, or painful conditions that could cause respiratory difficulty; a young age and compatible radiological findings reinforced suspicion.

Cats were classified into the traumatic group (T) if trauma was explicitly reported or suspected based on the identification of compatible lesions: confirmed facial trauma, pneumothorax, pneumomediastinum, rib fractures, hemothorax, or diaphragmatic hernia. Only traumatic pneumothoraxes were classified in this group, while those resulting from a tumor-related disruption of the respiratory system were included in the neoplastic group (N).

Cats were classified into the neoplastic group (N) if the final diagnosis was a thoracic mass (excluding abscesses, which were classified in the respiratory group [7]) or cancerous pleural effusion. Cardiac tumors were included in this group rather than in the cardiac group.

Cats were classified in the undiagnosed/unrelated group (U) if no final diagnosis could be established, either because the cat died before investigations could be performed or because the owners refused further diagnostic tests. This group also included cats whose diagnoses were unrelated to respiratory system lesions, such as severe abdominal pain.

### 2.5. Statistical Analyses

The qualitative variables analyzed included breed, sex, lifestyle (outdoor access or not), medical history, mucous membrane color, presence of open-mouth breathing, categorized respiratory rate (bradypnea, normopnea, tachypnea), presence of paradoxical breathing (thoracoabdominal asynchrony), categorized heart rate (bradycardia, normocardia, tachycardia), cardiac auscultation findings (heart murmur, gallop rhythm, arrhythmia, or inaudible heart sounds), pulmonary auscultation findings, presence and cause of pleural effusion, cause of dyspnea (respiratory, cardiac, traumatic, neoplastic), in-hospital mortality, and circumstances of death (natural death due to disease progression or euthanasia).

The quantitative variables analyzed included age, duration of dyspnea prior to presentation, and rectal temperature.

The variables were first analyzed for all cats combined and then for each group according to the cause of dyspnea (respiratory, cardiac, traumatic, or neoplastic) in order to evaluate the epidemiological, clinical, and prognostic differences between the groups.

Comparisons between groups were performed based on the formulated hypotheses and the nature of the data.

The statistical hypotheses associated with the comparisons were as follows: the null hypothesis stated that no difference existed between the groups for the parameter under study, whereas the alternative hypothesis postulated the existence of a difference between two groups for the parameter under investigation.

Statistical tests were selected according to the characteristics of the studied variables:

- For qualitative variables, comparisons between groups were performed using the Chi^2^ test. When the expected frequencies were too low (at least one expected frequency below 1 or more than 20% of expected frequencies below 5), Fisher’s exact test was applied.
- Quantitative data (age, etc.) did not follow a normal distribution (observed through heterogeneity in minimum and maximum values). Therefore, the results were summarized as medians and ranges (minimum and maximum values). Comparisons between groups were performed using the Kruskal–Wallis test. To control the type I error risk due to the high number of pairwise comparisons (k = 6), Dawns–Steel–Critchlow–Fligner pairwise comparisons were carried out.
- To evaluate whether the sex distribution significantly differed from a theoretically balanced distribution (50% males, 50% females), a two-tailed binomial test was used.

Calculations were performed using JAMOVI software, version 2.3.28.0. A *p*-value below 0.05 was considered statistically significant.

## 3. Results

Epidemiological data and medical history are summarized in Table 1; clinical presentations are summarized in Table 2; outcomes are summarized in Table 3.

### 3.1. Results for All Cats Combined

A total of 312 dyspneic cats meeting the inclusion criteria were treated over the 5-year period. The cause of dyspnea was identified in 258 cats, accounting for 83% of cases (*n* = 258/312), while no etiology could be determined in 54 cases (17%). The study focused exclusively on the 258 cats with an etiological diagnosis.

The 258 cats for which a cause of dyspnea was identified were categorized as follows:

- 33% (*n* = 84/258) had respiratory-origin dyspnea (Group R);
- 25% (*n* = 65/258) had cardiac-origin dyspnea (Group C);
- 21% (*n* = 55/258) had trauma-related dyspnea (Group T);
- 21% (*n* = 54/258) had neoplastic-origin dyspnea (Group N).

For the remaining 54 cats (Group U), 93% (*n* = 50/54) were cases in which the cause of dyspnea was not investigated. This was either because the cat died before diagnostic workup could be performed (in many cases, dyspnea was the clinical expression of impending death) or because owners declined investigations, primarily for financial reasons and/or due to the cat’s age. For the remaining four cats (7%), a cause of dyspnea was identified but was neither cardiac, respiratory, traumatic, nor neoplastic: two cats had upper urinary tract stones likely causing renal colic, one cat had bilateral nephromegaly, and one cat presented with severe anemia.

Thoracic radiographs were performed in 84% (*n* = 217/258) of the cats. POCUS was conducted in 42% (*n* = 108/258) and echocardiography in 17% (*n* = 43/258). All cats in this series underwent at least one imaging modality, either radiography or ultrasound, and cytological analysis was performed when indicated. Over the 5-year study period, POCUS became increasingly common.

Pleural effusion was identified in 112 out of 312 dyspneic cats (36% of the total). Among the 54 cats without a definitive etiological diagnosis (Group U), at least 12 (22%; *n* = 12/54) had pleural effusion. Among the 258 cats with an etiological diagnosis, pleural effusion was present in 39% (*n* = 100/258). Only 43% of cats with pleural effusion (*n* = 43/100) exhibited paradoxical breathing. The origin of the effusion was determined based on cytological analysis and, when indicated, FIP PCR testing. The 100 identified pleural effusions were categorized as follows:

- 38% (*n* = 38/100) were cardiac in origin;
- 26% (*n* = 26/100) were due to pyothorax;
- 23% (*n* = 23/100) were neoplastic;
- 9% (*n* = 9/100) were traumatic;
- 4% (*n* = 4/100) were associated with feline infectious peritonitis (FIP).

### 3.2. Results for Cats in Group R—Respiratory Origin

A respiratory origin was identified in 33% of the cats (*n* = 84/258), classified as group R.

**Figure 3:**
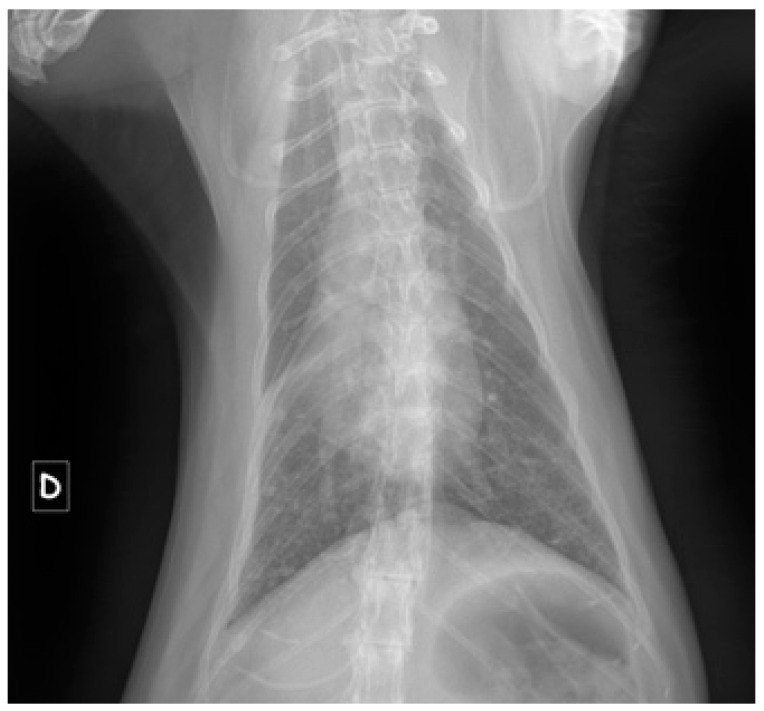
Frontal radiographic image of a cat with suspected feline asthma. The diagnosis of feline asthma was one of exclusion. A relatively young age, reported episodic coughing or dyspnea, expiratory effort, subtle radiographic lesions compared to the severity of symptoms, suggestive radiographic findings (donut-shaped opacities, pulmonary hyperinflation, atelectasis of the right (D) middle lobe), and a favorable response to treatment all supported this diagnosis.

Among the 84 cats in group R, 31% (*n* = 26/84) were diagnosed with pyothorax, while 25% (*n* = 21/84) had feline asthma (Figure 3). Bronchopneumonia was identified in 18% of cases (*n* = 15/84), and feline upper respiratory syndrome (coryza) was noted in 13% (*n* = 11/84). Additionally, 5% of cats (*n* = 4/84) presented with pleural effusion associated with feline infectious peritonitis (FIP). The remaining cats (*n* = 7/84) were diagnosed with tracheitis (two cases), chronic bronchitis, diaphragmatic pericardial hernia, spontaneous pneumomediastinum, granulomatous laryngitis, or laryngeal paralysis.

Within the group of cats diagnosed with pyothorax (*n* = 26/84), males represented 69% of cases (*n* = 18/26), while females accounted for 31% (non-significant, *p* = 0.076; *n* = 8/26). The median age of cats with pyothorax was 4.9 years (range: 8 months–16 years). Most cats with pyothorax, 92% (*n* = 24/26), were domestic shorthair cats. The remaining cats included one Sacred Birman and one Bambino. Regarding lifestyle, 85% of cats with pyothorax (*n* = 22/26) had mixed indoor–outdoor access, while 15% (*n* = 4/26) lived strictly indoors. Of the indoor-only cats, one lived alone, one cohabited with twelve other cats, and the remaining two lived with another cat, one of which also cohabited with a dog. Rectal temperature was recorded in 22 of the 26 cats with pyothorax. Hyperthermia was noted in only 32% (*n* = 7/22), hypothermia in 50% (*n* = 11/22), and normothermia in 18% (*n* = 4/22). Among cats with pyothorax, 46% (*n* = 12/26) survived the episode and were discharged from the hospital. Of the 54% of cats (*n* = 14/26) that died, half (*n* = 7/14) were euthanized, and the other half (*n* = 7/14) died of natural causes.

In the group of cats diagnosed with asthma (*n* = 21/84), males accounted for 52% (*n* = 11/21), and females represented 48% (non-significant, *p* = 0.827; *n* = 10/21). The majority, 90% of cases (*n* = 19/21), were domestic shorthair cats. One Sacred Birman and one Persian were also included. The median age of cats with asthma was 5.9 years (range: 9.8 months–16 years). Survival was favorable for 90% of cats (*n* = 19/21), while 10% (*n* = 2/21) were euthanized.

In the group of cats diagnosed with bronchopneumonia (*n* = 14/84), hypothermia was noted in 43% of cases (*n* = 6/14), normothermia in another 43% (*n* = 6/14), and hyperthermia in only 14% (*n* = 2/14).

### 3.3. Results for Cats in Group C—Cardiac Origin

A cardiac origin was identified in 25% of the cats (*n* = 65/258), representing Group C.

Among the 65 cats diagnosed with cardiac disease, 66% (*n* = 43/65) underwent echographic diagnosis of the cardiac lesion. Cardiac lesions identified in this subgroup are summarized in Table 4. For the remaining 34% (*n* = 22/65) of cats, cardiac disease was diagnosed based on radiographic evidence of cardiomegaly or a left atrium-to-aorta (LA/Ao) ratio greater than 1.55 measured via cardiac POCUS, without further investigation to determine the precise lesion.

Among cats with hypertrophic cardiomyopathy, 72% (*n* = 18/25) were males, while 28% (*n* = 7/25) were females, with a significant difference in sex distribution (*p* = 0.028). The median age of cats with HCM was 7.5 years (range: 10 months–18 years). Most cats in this subgroup, 92% (*n* = 23/25), were domestic shorthair cats, with one Siamese and one Persian cat also represented. Survival was achieved in 76% (*n* = 19/25) of the cats, while 24% (*n* = 6/25) did not survive. Among the non-survivors, 67% (*n* = 4/6) were euthanized, and 33% (*n* = 2/6) died naturally.

**Figure 4:**
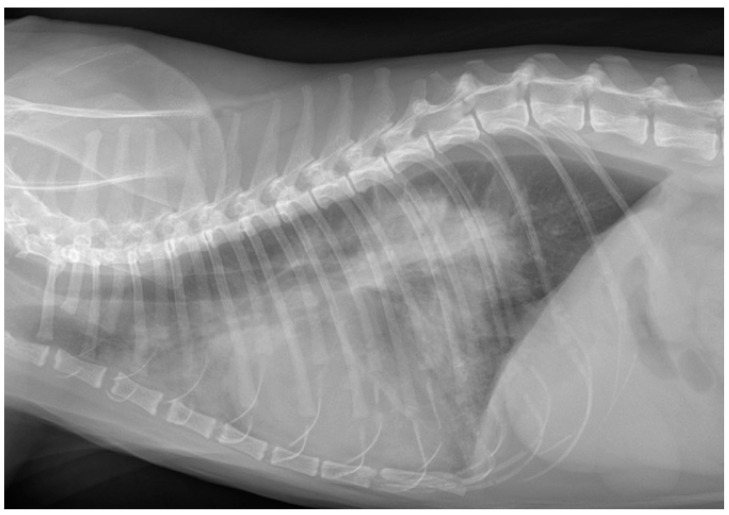
Pulmonary edema, pleural effusion, and cardiomegaly in a dyspneic cat. The cats in this series with congestive heart failure presented with pulmonary edema in 67% of cases, pleural effusion in 62% of cases, and both lesions simultaneously in 30% of cases.

Among the cats in Group C, a respiratory lesion explaining the dyspnea was identified in 94% (*n* = 61/65) of cases. Pulmonary edema was present in 67% (*n* = 41/61) of the cats, while 62% (*n* = 38/61) had pleural effusion. Both conditions occurred simultaneously in 30% (*n* = 18/61) of cases, but when pleural effusion was significant, pulmonary edema could not always be observed (Figure 4). In four cats, no pulmonary edema or pleural effusion was detected. One of these cats presented with aortic thromboembolism and respiratory crackles and died at admission. Two cats, previously diagnosed with congestive heart failure, were euthanized at admission. A fourth case involved a 28-day-old kitten with severe cardiomegaly, also euthanized at admission due to suspected congenital heart disease.

### 3.4. Results for Cats in Group T—Traumatic Origin

A traumatic origin was identified in 21% of the cats (*n* = 55/258), categorized as Group T.

Recent trauma was reported by owners in 65% of cases (*n* = 36/55), including road traffic accidents, falls from heights, and dog bites. The remaining 20% (*n* = 11/55) consisted of cats returning from outings with lesions strongly suggestive of trauma, such as skull fractures, facial fractures, rib fractures, femoral fractures, or pneumothorax. The estimated median time to dyspnea onset in Group T was 2 h (range: 1 h–21 days), with 84% (*n* = 46/55) of cats presenting with dyspnea within 24 h.

Among the cats in Group T with confirmed or strongly suspected trauma, diaphragmatic hernia was the most common lesion, observed in 28% (*n* = 15/55) of cats; pulmonary contusions alone were identified in 24% (*n* = 13/55), followed by traumatic pneumothorax in 22% (*n* = 12/55). Tracheal rupture, rib fractures, and pneumomediastinum were each reported in 4% (*n* = 2/55) of cats. Among the remaining nine cats (*n* = 9/55), five had facial trauma with upper airway obstruction caused by blood; one cat presented with a lead pellet in the mediastinum, while another had hemomediastinum; finally, two cats were euthanized without investigation of the lesion responsible for dyspnea.

The median age of cats with diaphragmatic hernia in this series was 1.6 years. Males were overrepresented, accounting for 73% (*n* = 11/15) of cases, whereas females represented 27% (*n* = 4/15). Paradoxical breathing was noted in 67% (*n* = 10/15) of cases. Surgery was performed in 80% of cases (*n* = 12/15), while the procedure was declined for financial reasons in 20% (*n* = 3/15). Among the cats that underwent surgery, one died during the operation, while the remaining eleven cats were discharged. Overall, 27% (*n* = 4/15) of cats with a diaphragmatic hernia died, including 75% (*n* = 3/4) due to financial limitations.

The median age of cats with traumatic pneumothorax was 1.2 years. Males represented 58% (*n* = 7/12) of cases, whereas females accounted for 42% (*n* = 5/12). Paradoxical breathing was noted in 50% (*n* = 6/12) of cases. In cats diagnosed with traumatic pneumothorax, 58% (*n* = 7/12) survived the episode, while 42% (*n* = 5/12) did not survive. Among the non-survivors, 60% (*n* = 3/5) were euthanized, and 40% (*n* = 2/5) died naturally.

### 3.5. Results for the Cats in Group N—Neoplastic Origin

A neoplastic origin was identified in 21% of cats (*n* = 54/258), placed in Group N.

The tumor type was identified in 69% of cats (*n* = 37/54) (Table 5). For the remaining 31% of cats (*n* = 17/54), the histological tumor type was not investigated.

**Figure 5:**
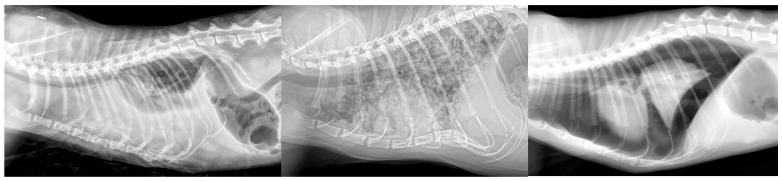
Neoplasia accounted for 21% of the causes of dyspnea in this series. The median age of the affected cats was 11 years. The lesions responsible for dyspnea varied: effusion (**on the left**), diffuse parenchymal involvement (**in the center**), and pneumothorax (**on the right**).

The lesions responsible for dyspnea varied: pleural effusion, diffuse parenchymal involvement, pneumothorax, etc. (Figure 5).

### 3.6. Comparison Between Groups

#### 3.6.1. Sex

Male cats represented 57% of the study population (*n* = 148/258) and were significantly more numerous than females (*n* = 110/258) (*p* = 0.021).

Males were significatively more represented than females in Group R (*p* = 0.006) and Group C (*p* = 0.046), but this was not the case for Group T (*p* = 0.281) and N (*p* = 0.076).

Among the cardiac cats (Group C), within the subgroup of cats with hypertrophic cardiomyopathy (HCM), 72% were male (*n* = 18/25) and 28% were female (*n* = 7/25), a statistically significant finding (*p* = 0.028).

#### 3.6.2. Age

Cats in Group T had a median age of 2 years and were significantly younger compared to cats in all other groups (N, C, and R) (*p* < 0.001).

Cats in Groups C and N had comparable ages (no significant difference), approximately 11 years; they were older than cats in Group R and cats in Group T (*p* < 0.001 for both).

#### 3.6.3. Lifestyle

The majority of cats (69%; *n* = 179/258) had access to the outdoors (*p* = 0.046).

Although the proportion of cats with outdoor access was higher in cats with pyothorax (85%; *n* = 22/26) compared to the group without pyothorax (68%; *n* = 157/231), this difference was not statistically significant (*p* = 0.08).

#### 3.6.4. Medical History

The T group, which included cats that had experienced trauma, had the highest proportion of acute dyspnea (<24 h) (84%) and was significantly different from all other groups (R, C, and N; *p* = 0.004, *p* = 0.019 and *p* < 0.001 respectively).

The N group, which included cats with cancer, had the lowest proportion of acute dyspnea (43%) and was significantly different from groups R, C, and T with *p* = 0.037, *p* < 0.001, and *p* < 0.001, respectively.

In the N group, 24% of cats (*n* = 13/54) had respiratory signs lasting for more than 7 days, compared to 6% (*n* = 5/84), 3% (*n* = 2/65), and 4% (*n* = 2/55) for the respiratory, cardiac, and traumatic groups, respectively. Statistically, the N group had the highest proportion of cats with dyspnea lasting more than 7 days compared to all other groups (R, C, and T), with *p* = 0.003, *p* < 0.001, and *p* = 0.002, respectively. No significant difference was found between groups R, C, and T.

Symptoms unrelated to dyspnea (dysorexia, weight loss, reduced water intake, polydipsia) were reported across all groups, often with similar proportions.

Dysorexia was significantly less frequently reported in cats belonging to the T group (traumatic) compared to cats in the other groups (R: *p* > 0.001, C: *p* = 0.025, and N: *p* = 0.001), since dyspneic cats with trauma were admitted for consultation rapidly (on the same day in 83% of cases). Regarding dysorexia, there was no significant difference between the R, C, and N groups.

Weight loss was significantly more frequent in the N group, reported by 31% of owners, with statistically significant differences compared to the R group (*p* = 0.001), the C group (*p* = 0.020), and the T group (*p* < 0.002).

Cough was significantly more frequent in the R group than in the C group (*p* = 0.005) and the T group (*p* < 0.002). There was no significant difference between groups R and N (*p* = 0.220), nor between C and N (*p* = 0.146) or C and T (*p* = 0.139).

#### 3.6.5. Clinical Presentation

(a). Temperature

With hypothermia present in 82% of cases (*n* = 45/55), cardiac cats were significantly more likely to be hypothermic compared to cats in the other groups (R, T, and N), with *p* < 0.0001, *p* = 0.025, and *p* = 0.011, respectively. The median rectal temperature in the C group was significantly lower than that in the R group (*p* < 0.001) and in the N group (*p* = 0.047) but not in the T group (*p* = 0.242).

The R group had a lower proportion of hypothermic cats compared to the other groups. The median rectal temperature was significantly higher in the R group (median 38.4 °C) compared to that in the C group (*p* < 0.001), the T group (*p* = 0.017), and the N group (*p* = 0.024). The R group had a significantly higher frequency of hyperthermia compared to the C group (*p* < 0.001), the T group (*p* = 0.005), and the N group (*p* = 0.005); there was no significant difference in hyperthermia between the C, T, and N groups.

(b). Respiratory assessment

Regarding respiratory rate, the proportion of cats with tachypnea was higher in the R and C groups than in the T and N groups (*p* < 0.001). There was almost no normopnea observed in the R group or the C group, with proportions of 3% and 5%, respectively.

Cyanosis was most frequently observed in the C group, where it was present in 16% of cats (*n* = 10/63), compared to only 4% of cats in the R group. Cats in the C group were significantly more likely to exhibit cyanosis than those in the R group (*p* = 0.017), but no significant difference was observed between cats in the N group (*p* = 0.321) and the T group (*p* = 0.139).

(c). Cardiac assessment

Neither tachycardia nor bradycardia was observed significantly more frequently in any particular group when comparing all groups pairwise.

Bradycardia was more common than tachycardia in dyspneic cats in this series (58/234 cats vs. 33/234 cats, respectively; *p* = 0.006).

A gallop rhythm was identified on admission in only 3% of cases (*n* = 7/246), all of which belonged to the C group.

As expected, the C group had a significantly higher proportion of heart murmurs compared to Groups R (*p* = 0.021), T (*p* < 0.001), and N (*p* = 0.015). No significant differences were found between Groups R, T, and N.

#### 3.6.6. Outcome

Statistically, the respiratory, cardiac, and traumatic groups did not show significant differences in survival rates (*p* > 0.05). In contrast, the neoplasia group had a significantly lower survival rate than all other groups (*p* < 0.001).

## 4. Discussion

This series represents the largest study examining the causes of acute dyspnea in cats, regardless of origin. The study conducted by Swift et al. in Liverpool, published in 2009, included 90 feline cases [1]. The RAPID CAT study by Dickson et al., which served as an inspiration for the present study [2], was carried out in the United Kingdom and published in 2018, and it included 101 cats recruited from nine veterinary centers, of which 92 cats received a definitive diagnosis. In comparison, the present study encompasses nearly three times the number of cases analyzed in these earlier investigations.

The primary objectives of these three studies were to investigate the causes of acute dyspnea in a population of urban cats and to identify clinical indicators that differentiate cardiac from non-cardiac causes.

### 4.1. Discussion on Epidemiology

#### 4.1.1. Breed

In the absence of a reference population, it is not possible to establish a breed predisposition to dyspnea, regardless of the identified cause. Despite known breed predispositions for certain cardiomyopathies, Group C in our study population was predominantly composed (90%) of domestic shorthair cats. This finding is consistent with the literature, which, however, does not highlight the overwhelming proportion of domestic shorthair cats observed here [8]. Many publications tend to focus on specific purebred populations, suggesting an elevated risk within these breeds while inadvertently implying a reduced susceptibility in domestic shorthair cats. The underrepresentation of purebred cats in our cohort likely reflects the broader demographics of the general feline population, where domestic shorthair cats are overwhelmingly prevalent. Pairwise comparisons between groups showed that the proportions of domestic shorthair cats were statistically similar across all groups.

#### 4.1.2. Sex

Male cats accounted for 57% of the study population, representing a significantly higher proportion compared to females. However, in the absence of a reference population, it is not possible to establish a definitive sex predisposition, as the baseline distribution of male and female cats in the general feline population of our metropolitan area remains unknown.

Males were significatively more represented than females in Group R and Group C, but this was not the case for Group T and N.

Within the cardiac group, male cats were particularly overrepresented among those diagnosed with hypertrophic cardiomyopathy, a finding consistent with previously published data [8,9,10].

#### 4.1.3. Age

The age distribution was multimodal, with three age classes according to the causes of dyspnea (Table 6).

Cats in Group T had a median age of 2 years and were significantly younger compared to those in all other groups. A similar observation was made in the British study by Dickson et al. [2], where the median age of traumatized cats was 3 years. It is logical to suggest that younger cats are more likely to suffer trauma.

Cats in Groups C and N had comparable ages, approximately 11 years; they were older than cats in Group R and cats in Group T.

Age appears to be the most determining parameter for predicting the origin of dyspnea in cats. If a cat had not experienced trauma and was young (approximately 6 years old), there was a higher probability that its dyspnea was of respiratory origin. In contrast, for older cats (around 11 years), there was a higher probability that the dyspnea was either cardiac or neoplastic in origin. While age was a useful indicator in differentiating between respiratory and non-respiratory causes of dyspnea, it was not sufficient to distinguish between cardiac and neoplastic origins. However, hypothermia was more commonly observed in cats with cardiac conditions than in those with neoplasia, providing an additional diagnostic clue (see below).

In this series, cardiac cats had a median age of 11 years, similar to the findings of Dickson et al. [2], where the median age of cardiac cats was 11.6 years. In contrast, the study by Swift et al. [1] reported younger cardiac cats, with a median age of 6.1 years.

Cats with pyothorax in this series had a median age of 4.9 years, which is consistent with the study by Waddell et al. [11], including 80 cats with pyothorax, where the mean age was 3.8 years.

Cats with asthma had a median age of 5.9 years. This value is consistent with the literature, which reports a median age between 4 and 5 years [12].

Cats with neoplasia had a median age of 11.7 years. Among cats with mediastinal lymphoma, 60% (*n* = 6/10) were 1 year old or younger. In most studies on mediastinal lymphoma, the median age of affected cats ranges from 2 to 4 years. In the study by Fabrizio et al. [13], which examined 55 cases of mediastinal lymphoma, the median age was 3 years with a bimodal age distribution, with a major peak at 1 year and a secondary peak at 8 years.

#### 4.1.4. Lifestyle

The majority of cats in this study (69%) had access to the outdoors. However, in the absence of a reference population, it is not possible to determine whether outdoor access constitutes a significant risk factor for the development of dyspnea. It is reasonable to assume an association in the traumatic group (Group T), where the primary causes of injury were road traffic accidents and dog bites.

No significant difference in outdoor access was observed between cats with and without pyothorax. Among the four cats diagnosed with pyothorax that did not have outdoor access, one lived in a single-cat household, another cohabited with twelve other cats, and the remaining two lived with one other cat—one of which also shared the household with a dog. These findings align with those reported by Waddell et al. [11], who found that outdoor access was not significantly associated with an increased risk of pyothorax. However, their study did reveal that cats living in multi-cat households were 3.8 times more likely to develop pyothorax compared to those living alone.

### 4.2. Discussion on Clinical Presentation

#### 4.2.1. Medical History

The delay between the onset of dyspnea and presentation was most often short: respiratory distress had been observed for less than 24 h before presentation in 63% of cats. This can easily be explained by the dramatic nature of severe dyspnea, which raises concerns about imminent death. There is a recruitment bias, as only cases admitted to the emergency and intensive care service were included in this study.

Cats in the T group, which included those that had experienced trauma, were presented more rapidly for veterinary care compared to those in all other groups.

Cats in the N group, which included those with cancer, were less likely to present with acute dyspnea compared to the other groups. When dyspnea persisted for more than 24 h, it was more likely to be associated with neoplasia.

Cats in the N group were more likely to exhibit respiratory signs lasting longer than 7 days compared to the other groups, while no significant differences were observed between the respiratory, cardiac, and traumatic groups.

The study by Dickson et al. [2] did not report any differences between groups regarding the duration of dyspnea before presentation; this may be explained by the smaller sample size in that study.

Non-dyspneic symptoms such as dysorexia, weight loss, and cough were reported across all groups with varying frequencies. Dysorexia was less frequently observed in the traumatic group due to rapid presentation, while weight loss was more common in the neoplasia group. No significant differences were found for dysorexia between the respiratory, cardiac, and neoplastic groups.

A history of coughing was reported in only 11% of cardiac cats in this series, which is two times lower than in the series by Dickson et al. [2], where cough was reported in 25% of cardiac cats. Cough was not predictive of a particular condition: approximately 10% of cats in the cardiac group (C) presented with cough, compared to 20% of cats with cancer (N group) and 30% of cats in the respiratory group (R). Cough was reported by 67% of owners of cats considered asthmatic; while this frequency is high, the literature reports even higher frequencies, with 90% [14] and even 95% [15] of asthmatic cats presenting with cough. Cough was reported in 20% of cats with cancer, which is almost half the proportion observed in the study by Aarsvold et al. [7] (37%) and the study by Dickson et al. [2] (40%).

#### 4.2.2. Clinical Presentation

(a). Temperature

Hypothermia was a consistent finding in cardiac cats, occurring in 82% of cases, with a median rectal temperature of 36.7 °C. Cardiac cats were significantly more likely to be hypothermic compared to cats in the other groups. This observation was also reported in the series by Dickson et al. [2]. As highlighted by Dickson et al. [2], a rectal temperature exceeding 40 °C made the diagnosis of congestive heart failure highly unlikely. In our series, only 1 cardiac cat among the 55 cardiac cats exhibited hyperthermia on admission.

The median rectal temperature was significantly higher in the R group compared to that in the other groups. This was associated with a lower proportion of hypothermic cats and a significantly higher frequency of hyperthermia, likely due to the presence of pyothorax and bronchopneumonia cases in this group.

Among the cats diagnosed with pyothorax, bronchopneumonia, or FIP, only 25% presented with hyperthermia. Overall, hyperthermia exceeding 39.2 °C was observed in 22 of the 217 cats with available temperature data. An infectious process was confirmed in 45% of these hyperthermic cats (seven with pyothorax, two with bronchopneumonia, and one with FIP), while 56% had no confirmed infectious cause. In this cohort, hyperthermia in dyspneic cats proved to be neither a sensitive nor a specific indicator of infection. Consequently, the algorithm proposed by Dickson et al. [2], which prioritizes an infectious origin in cases of hyperthermia, would not be sufficiently reliable when applied to the feline population in this study.

(b). Respiratory assessment

Median respiratory rates were identical or nearly identical across all groups, averaging 50 breaths per minute.

Cyanosis was observed in only 9% of dyspneic cats in this cohort (*n* = 21/247), a surprisingly low figure considering the severity of respiratory distress documented. This finding may be attributed to the subjective nature of cyanosis assessment, which was not consistently corroborated by pulse oximetry (SpO₂) measurements, as efforts were made to minimize stress in these critically ill patients. Cyanosis was most frequently noted in the cardiac group (C group), where it was present in 16% of cases, compared to only 4% in the respiratory group (R group).

Paradoxical breathing was identified in 37% of the cats. It was present in 67% of cats with diaphragmatic hernia, 43% of those with pleural effusion, and 50% of cats with traumatic pneumothorax. However, paradoxical breathing was not universally observed in these conditions. Conversely, 38% of cats exhibiting paradoxical breathing had no evidence of diaphragmatic hernia, pleural effusion, or traumatic pneumothorax, suggesting the absence of a confirmed mechanical impairment of diaphragmatic function. These observations underscore the limitations of clinical assessment in severely dyspneic cats. Similar limitations were reported in two other French studies: one involving 125 cats with paradoxical breathing [16] and another including 380 cats with pleural effusion [17]. A plausible explanation for these findings is the potential difficulty in distinguishing paradoxical breathing from costal retraction (abnormal rib movement) or pronounced abdominal effort (excessive contraction of the abdominal musculature).

(c). Cardiac assessment

Only 25% of severely dyspneic cats admitted to our emergency service were cardiac. This proportion is lower than the 38% reported in the study by Swift et al. [1] and considerably lower than the 65% observed by Dickson et al. [2]. This corroborates our initial impression that cardiac disease was not diagnosed in nearly two-thirds of dyspneic cats admitted to our emergency service.

The median heart rate of cats was the same across the four groups, at 180 bpm.

Tachycardia was not observed significantly more frequently in any particular group. This observation is counterintuitive, as cardiac cats are expected to have high sympathetic tone to maintain cardiac output. As Dickson et al. [2] remarked for their series, it is surprising to find that the majority of dyspneic cats—86% in the present series—had a normal or low heart rate, meaning they were not tachycardic, despite the stress of dyspnea, the journey to the hospital, and the clinical examination.

Bradycardia was not observed significantly more frequently in any particular group. Counterintuitively, bradycardia was more common than tachycardia in dyspneic cats in this series. This corroborates the findings of Smith et al. [18] in a study of 51 cardiac cats with clinical signs: while tachycardia (<200 bpm) was expected due to cardiac failure and presumed sympathetic activation, it was observed in only 25% of cases, whereas bradycardia (<160 bpm) was seen in 18% of cats. This may reflect specific features of the feline autonomic nervous system or the activation of compensatory mechanisms that differ from those seen in dogs. Similar observations had been made in earlier studies [19].

Gallop sounds appeared to be highly specific to cardiac disease (all of which cases belonged to the C group) but were identified on admission in only 12% of cardiac cats. These figures are lower than those reported by Goutal et al. [20], who noted gallop sounds in 62% of cardiac cats in their series. Dickson et al. [2] considered gallop sounds a key element in their algorithm for identifying cardiac disease; however, gallop sounds were present in only 23% of cats in their series. We believe that the low detection rate of gallop sounds in our series may be due to a lack of awareness among frontline veterinarians regarding the recognition of this additional heart sound. If a senior veterinarian identified a gallop rhythm the following day, it might not have been corrected in the admission records. It is also possible that gallop sounds were overdiagnosed in other studies.

In this series, heart murmurs were audible in only 31% of cardiac cats, meaning that 69% of cardiac cats had no detectable murmur. This finding corroborates observations from previous studies [1,2,18,20]. The detection of heart murmurs is therefore a poorly sensitive indicator of cardiac disease in cats, which contrasts sharply with dogs, in which murmurs are almost always audible in cases of decompensated heart disease. Heart murmurs can be challenging to detect in dyspneic cats due to tachycardia, pleural effusion, or additional pulmonary sounds; therefore, their presence or absence cannot be used as the sole diagnostic criterion. As expected, the C group had a significantly higher proportion of heart murmurs compared to the other groups. So, in dyspneic cats in this series, the detection of a heart murmur was suggestive of cardiac disease (with 31% of cardiac cats had a murmur), but this symptom was not a reliable marker for predicting a cardiac origin; indeed, a murmur was noted in 10% of non-cardiac cats (*n* = 19/187). In the study by Dickson et al. [2], only 23% of cardiac cats had a murmur, whereas a murmur was audible in 47% of cats in the R group.

(d). Pleural effusions

Pleural effusion was identified in 39% of cases (*n* = 100/258), making it a major cause of severe dyspnea in cats. Given its frequency and its severity, it is essential to detect pleural effusion promptly, ideally bedside using thoracic POCUS or alternatively with radiography if the cat can tolerate the procedure. This rapid detection allows for early fluid evacuation through thoracocentesis, which provides relief and allows for analysis to identify the underlying cause.

The main causes of pleural effusion were cardiac disease, pyothorax, and thoracic neoplasia. The proportion of pleural effusions of cardiac origin in our series (38%) was similar to that reported in other recent studies [21,22,23] (Table 7).

Similarly, the proportion of pleural effusions of neoplastic origin was comparable across the four studies (around 25%). The proportion of effusions associated with FIP ranged from 1% to 9%.

In this series, 26% of pleural effusions were diagnosed as pyothorax, a higher proportion compared to other studies. This discrepancy may be explained by differences in the lifestyles of the studied populations.

Among the dyspneic cats in this series where cardiac disease was diagnosed, the respiratory lesion explaining dyspnea was as often pulmonary edema (67%) as pleural effusion (62%), with both lesions coexisting in 30% of the cats. These figures are consistent with the study by Goutal et al. [20], which examined radiographs of 40 cats with congestive heart failure.

No chylothorax was identified during this five-year period. This may be coincidental, as cases were recorded in previous years as well as the year following the study period.

### 4.3. Discussion on the Causes

In this series, the respiratory group (R) was the most represented, accounting for 33% of dyspneic cats (*n* = 84/258). Cardiac cats (C) ranked second, representing 25% of cases (*n* = 65/258), followed by cats with trauma (21%) and those with cancer (21%), both at equal proportions. So, this study does not confirm the overwhelming majority of cardiac cats diagnosed in the study by Dickson et al. [2], which reported 65% cardiac cats (*n* = 60/92) but only 16% (*n* = 15/92) with a respiratory cause, 11% (*n* = 10/92) with a neoplastic cause, and 8% (*n* = 7/92) with a traumatic origin. Compared to the British study, the French study identified proportionally far fewer cardiac cats (*p* < 0.0001), significantly more cats with respiratory involvement (*p* = 0.003), a higher number of trauma cases (*p* = 0.003), and an equivalent proportion of cats with cancer (*p* = 0.032). This discrepancy could be attributed to geographical, epidemiological, or even methodological variations between the two studies. For instance, in the series by Dickson et al. [2], dyspnea cases of traumatic origin were proportionally lower (8%; *n* = 7/92) than in our series, as five cases (out of the sample of 101 cats) of traumatic dyspnea were excluded based on evidence of observed trauma or visible injuries, whereas in our series, such observations constituted an inclusion criterion for this group.

The rate of undiagnosed cases in our series was 17%. This was not due to difficulties in establishing a diagnosis but rather to circumstances unfavorable for investigations: cats presented in a moribund state, often old, with known histories of severe illnesses, or cases where owners could not or did not wish to incur expenses. This rate was comparable to that reported in the study by Dickson et al. (14%).

Among the 65 cats diagnosed as cardiac, 66% had a precise echographic diagnosis of the cardiac lesion; in 93% of cases, it was a cardiomyopathy. Among the 40 cases of cardiomyopathy, 62% were hypertrophic cardiomyopathies.

Pyothorax in this series accounted for 31% of the cats in group R, 10% of all dyspneic cats, and 26% of cats with pleural effusion. Only 32% of cats with pyothorax were hyperthermic; hypothermia was more common, occurring in 50% of cases. Nearly half (46%) of the cats presenting with pyothorax survived.

A definitive diagnosis of feline asthma is challenging, as it is primarily a diagnosis of exclusion. A relatively young age (5.9 years in this study), cough (although less frequently observed in this series) or episodic dyspnea reported by owners, expiratory effort, subtle radiographic lesions disproportionate to symptom severity, or suggestive radiographic findings (donut-shaped opacities, pulmonary hyperinflation, right middle lung lobe atelectasis) and a favorable treatment response guided this diagnosis. Cats considered asthmatic had the best prognosis in this series, with a survival rate of 90%; the two deceased cats were euthanized at the owners’ request.

This series recorded nearly three times as many traumatized cats (31%; *n* = 55/258) as the RAPID CAT study by Dickson et al. [2], which reported only 8% (*n* = 7/92). In addition to the recruitment bias mentioned earlier, the lifestyle of the cats in the RAPID CAT study, which was not described, might explain reduced exposure to risk. About a quarter (27%) of dyspneic cats following trauma had diaphragmatic hernias.

The proportion of dyspnea due to neoplasia (21%) was similar to that reported in the study by Swift et al. [1] and twice as high as in the study by Dickson et al. [2], but the difference was not statistically significant. As in the study by Swift et al. [1], the most commonly identified tumor in our study was lymphoma, with the majority being mediastinal lymphomas.

### 4.4. Discussion on the Outcome

The short-term survival rate—defined as the proportion of cats discharged from hospitalization—was 56%. Put less optimistically, nearly one out of two dyspneic cats did not survive hospitalization (44%). Natural deaths accounted for 28% of fatalities, while euthanasia represented 72%.

The overall survival rate was slightly lower than that reported in the study by Swift et al. [1] (56% vs. 63%). However, their study included only referred cases, introducing a possible recruitment bias, as severely dyspneic cats might have been euthanized by the primary care veterinarian. Additionally, only 58% of cats in the Swift et al. study were admitted as emergencies.

Cats with dyspnea of purely respiratory origin had the highest survival rate (70%), followed by cardiac cats with a survival rate of 63% and traumatized cats with a survival rate of 60%. Only 22% of cats with neoplasia survived the initial episode, with euthanasia being chosen in 90% of deaths due to their poor prognosis. The neoplasia group exhibited a markedly lower survival rate compared to all other groups, while no significant differences in survival rates were observed among the respiratory, cardiac, and traumatic groups.

**Figure 6:**
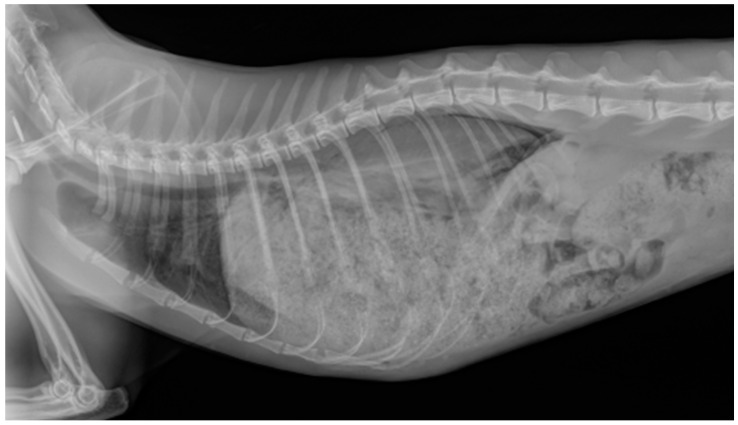
Diaphragmatic hernia with stomach displacement into the thoracic cavity. About a quarter of cats that were dyspneic due to trauma had a diaphragmatic hernia. The prognosis was good after surgery (90% survival), but 30% of owners declined the surgical procedure.

Among traumatized cats, those with diaphragmatic hernia that were able to undergo surgery had a good recovery rate in this series, with 92% survival, consistent with previously published data [24] (Figure 6).

**Figure 7:**
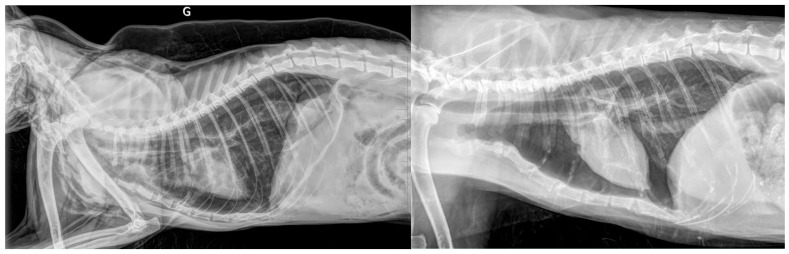
Traumatic pneumothorax in two cats. The assessment of dyspneic cats with pneumothorax, for whom an accident had been observed (case in **the left image**) or broken ribs were detected (case in **the right image**), led to the conclusion of a traumatic cause. Although traumatic pneumothorax is relatively easy to manage, it was associated with a high in-hospital mortality rate in this series (42%) due to associated injuries, which sometimes resulted in requests for euthanasia.

The in-hospital mortality rate for traumatized cats appears high (40%). It is surprising to observe that 42% of traumatic pneumothorax cases resulted in death, despite the fact that this condition is relatively straightforward to manage. In reality, these cats did not die from their pneumothorax; 45% of the deceased traumatized cats were euthanized. Among these 10 cats, 3 had diaphragmatic hernia that the owners declined to have surgically corrected, 1 had cranial trauma with a skull fracture, another had cranial trauma without fracture, and a 16-year-old cat in shock had an associated femoral fracture. Thus, pneumothorax is not inherently severe, but it is often associated with severe and costly injuries (Figure 7).

For cardiac cats (C) and cats with cancer (N) that survive the initial episode, it is important to keep in mind that the progression of their disease will often be fatal within relatively short timeframes. In this regard, even though their short-term survival rate was only 50%, traumatized cats and those with pyothorax that were discharged from the hospital had a better long-term survival outlook.

### 4.5. Limitations of the Study

This study examines only a subset of cats presenting with dyspnea, namely the most severely affected cases admitted to the emergency service. A recruitment bias therefore exists, as less severely affected cats may have been presented to other departments (internal medicine, imaging, cardiology). As a university center, the emergency and critical care service receives both its own clients and referred cases. Some cases are not explicitly referred but are redirected during nighttime or weekends to the emergency and critical care unit.

The limitations of this study are primarily linked to its retrospective nature. Certain data may have been missing, although this was rarely the case, as significant care was taken in information collection. Since the cost of diagnosis was borne by the owners, exhaustive investigations were not always possible. The primary goal was to obtain useful information to implement beneficial measures for the cats. Radiography remained the most commonly used diagnostic tool in this series. While POCUS became more widely adopted during the study period, its role deserves even greater emphasis in the assessment of dyspneic cats [4]. Similarly, cardiac biomarker testing warrants increased attention, provided the cat’s condition allows for blood sampling.

Some evaluated parameters, such as the respiratory curve, mucous membrane color, or the detection of a gallop sound, are subjective. The risk of differing interpretations for the same clinical presentation was exacerbated by the multiple clinicians conducting admission examinations. Although cats were re-evaluated by senior veterinarians within 12 h, symptoms observed after stabilization may have differed from those noted at admission—this was particularly true for pleural effusions before and after thoracocentesis.

## 5. Conclusions

This retrospective study demonstrates that the four major categories of causes (respiratory, cardiac, traumatic, and neoplastic) were represented in comparable proportions, each accounting for approximately one-quarter of cases. This contrasts with the British RAPID CAT study, where cardiac cats represented nearly two-thirds of dyspneic cases.

A comparison of the different groups (R, C, T, and N), examining factors such as epidemiology, history, and clinical presentation, identified very few parameters that could guide the identification of a specific cause of severe dyspnea in cats prior to confirmatory diagnostic tests. Among these, age was significantly associated with the underlying cause of dyspnea. Young cats, around 2 years old, presenting with dyspnea were more likely to have experienced trauma. In the absence of trauma, younger cats (around 6 years old) were more likely to exhibit dyspnea of respiratory origin, whereas older cats (around 11 years old) had a higher likelihood of dyspnea resulting from cardiac or neoplastic conditions. Age alone was not sufficient to differentiate between cardiac and neoplastic causes. Cats with cardiac-related dyspnea were more frequently hypothermic compared to those with neoplastic origins.

Some findings challenge preconceived ideas: hyperthermia was associated with a confirmed infectious process in only 45% of cases; paradoxical breathing was present in only 62% of pleural conditions; 38% of cats with paradoxical breathing did not have pleural involvement; a gallop sound was audible in only 12% of cardiac cats; and a cardiac murmur was not a reliable marker of cardiac abnormality, as its absence did not exclude a cardiac cause. Observations in this series suggest that the triage algorithm proposed by the British RAPID CAT study based on rectal temperature, the presence of a gallop sound, and heart and respiratory rates would not have been applicable to our cat population.

Regarding short-term prognosis, it can be considered poor, as nearly half of the cats did not survive hospitalization. The prognosis was particularly poor for cardiac and neoplastic cats, as many who survived hospitalization were likely to succumb to their condition in a relatively short timeframe. Conversely, medium- and long-term prognosis was more favorable for cats with respiratory or traumatic conditions that were discharged. Immediate prognosis was worse for cats with cancer, not because of the natural progression of the disease but due to more frequent decisions to euthanize.

Despite the usual importance clinicians place on semiology, which provides valuable and often overlooked information, it appears that in severely dyspneic cats, clinical presentation alone does not allow practitioners to determine a specific cause. This underscores the importance of non-invasive examinations such as POCUS, which has become a routine tool in veterinary emergency medicine and serves as an extension of the clinical examination that should become systematic in this context. This emphasis on POCUS should not diminish the value of radiography or the utility of cardiac biomarker testing, provided the cat’s condition allows for these tests.

## Figures and Tables

**Table 1:** Signalment and medical history of 258 cats that were admitted with severe dyspnea to a French emergency service where a diagnosis was reached.

Category	All Cats Combined	Respiratory (R)	Cardiac (C)	Trauma (T)	Neoplasia (N)
Number of cases (%)	258/312 (83%)	84 (33%)	65 (25%)	55 (21%)	54 (21%)
Breed (% domestic shorthair cats)	239 (93%)	79 (94%)	58 (89%)	52 (95%)	50 (93%)
Gender (% male cats)	148/258 (57%)	55/84 (65%)	41/65 (63%)	32/55 (58%)	20/54 (37%)
Median age (years)	7.7 (0.04–19.02)	6	11	2	11.7
Median duration of dyspnea (h)	24	24	24	2	36
Duration of dyspnea (<24 h)	162/258 (63%)	51/84 (61%)	42/65 (65%)	46/55 (84%)	23/54 (43%)
Duration of dyspnea (24 h–7 d)	74/258 (29%)	28/84 (33%)	21/65 (32%)	7/55 (13%)	18/54 (33%)
Duration of dyspnea (>7 d)	22/258 (9%)	5/84 (6%)	2/65 (3%)	2/55 (4%)	13/54 (24%)
Cough	45/258 (17%)	25/84 (30%)	7/65 (11%)	2/55 (4%)	11/54 (20%)
Wheeze	17/258 (7%)	8/84 (10%)	3/65 (5%)	2/55 (4%)	4/54 (7%)
Trauma	36/258 (14%)	0/84 (0%)	0/65 (0%)	36/55 (65%)	0/54 (0%)
Weight loss	35/258 (14%)	8/84 (10%)	9/65 (14%)	1/55 (2%)	17/54 (31%)
Dysorexia	115/258 (45%)	45/84 (54%)	28/65 (43%)	13/55 (24%)	29/54 (54%)
Reduced water intake	38/258 (15%)	11/84 (13%)	8/65 (12%)	7/55 (13%)	12/54 (22%)
Polydipsia	10/258 (4%)	4/84 (5%)	5/65 (8%)	0/55 (0%)	1/54 (2%)

**Table 2:** Clinical examination findings in 258 cats that were admitted with severe dyspnea to a French emergency service where a diagnosis was reached.

Category	All Cats Combined	Respiratory (*n* = 84)	Cardiac (*n* = 65)	Trauma (*n* = 55)	Neoplasia (*n* = 54)
Temperature, median (min–max)	37.6 °C (32.0–40.8)	38.4 °C (33.8–40.8)	36.7 °C (32.6–40.7)	37.6 °C (32.0–39.5)	37.6 °C (32.0–39.9)
Hypothermia	124/217 (57%)	27/75 (36%)	45/55 (82%)	27/42 (63%)	25/45 (56%)
Normothermia	72/217 (33%)	31/75 (41%)	9/55 (16%)	14/42 (33%)	18/45 (40%)
Hyperthermia	22/217 (10%)	17/75 (23%)	1/55 (2%)	2/42 (5%)	2/45 (4%)
Pink mucous membranes	162/247 (66%)	64/81 (79%)	35/63 (56%)	33/51 (65%)	30/52 (58%)
Pale mucous membranes	63/247 (26%)	13/81 (16%)	18/63 (29%)	15/51 (29%)	17/52 (33%)
Cyanosis	21/247 (8%)	3/81 (4%)	10/63 (16%)	3/51 (6%)	5/52 (10%)
Mouth breathing	52/258 (66%)	18/84 (21%)	12/65 (18%)	14/55 (25%)	8/54 (15%)
Paradoxical breathing	95/258 (37%)	34/84 (40%)	18/65 (28%)	21/55 (38%)	22/54 (41%)
Heart rate, median (min–max)	180 bpm	180 bpm (100–260)	180 bpm (64–250)	180 bpm (112–260)	180 bpm (80–260)
Bradycardia	58/235 (25%)	18/81 (22%)	11/55 (20%)	13/50 (26%)	16/49 (33%)
Normocardia	144/235 (61%)	55/81 (68%)	33/55 (60%)	28/50 (56%)	28/49 (57%)
Tachycardia	33/235 (14%)	8/81 (10%)	11/55 (20%)	9/50 (18%)	5/49 (10%)
Murmur	40/246 (16%)	12/83 (14%)	18/59 (31%)	3/52 (6%)	7/52 (13%)
Gallop	7/246 (3%)	0/83	7/59 (12%)	0/52	0/52 (0%)
Arrhythmia	4/246 (2%)	0/83	2/59 (3%)	0/52	2/52 (4%)
Inaudible cardiac sounds	40/246 (16%)	11/83 (13%)	13/59 (22%)	7/52 (13%)	9/52 (17%)
Respiratory rate, median (min–max)	50 mpm	50 mpm (28–150)	50 mpm (24–160)	50 mpm (8–100)	48 mpm (4–160)
Bradypnea	2/244 (<1%)	0/82	0/61	1/50 (2%)	1/51 (2%)
Normopnea	26/244 (10%)	3/82 (4%)	3/61 (5%)	11/50 (22%)	9/51 (18%)
Tachypnea	216/244 (89%)	79/82 (96%)	58/61 (95%)	38/50 (76%)	41/51 (80%)
Abnormal pulmonary auscultation	159/242 (66%)	57/79 (72%)	39/61 (64%)	29/49 (59%)	34/53 (64%)

**Table 3:** Outcomes in 258 cats that were admitted with severe dyspnea to a French emergency service where a diagnosis was reached.

Outcome	All Cats Combined	Respiratory (R)	Cardiac (C)	Trauma (T)	Neoplasia (N)
Survival	145/258 (56%)	59/84 (70%)	41/65 (63%)	33/55 (60%)	12/54 (22%)
Death	113/258 (44%)	25/84 (30%)	24/65 (37%)	22/55 (40%)	42/54 (78%)
Euthanized	81/113 (72%)	16/25 (64%)	17/24 (71%)	10/22 (45%)	38/42 (90%)
Natural death	32/113 (28%)	9/25 (36%)	7/24 (29%)	12/55 (55%)	4/42 (10%)

**Table 4:** Cardiac lesions identified in cats with heart disease.

Diagnosis	Count (*n* = 43/65)	Percentage (66%)
Hypertrophic cardiomyopathy	25	58%
Restrictive cardiomyopathy	8	19%
Unclassified cardiomyopathy	5	12%
Dilated cardiomyopathy	2	5%
Congenital heart disease	2	5%
Endocarditis associated with sepsis	1	2%

**Table 5:** Types of tumors identified in group N cats (*n* = 37/54).

Tumor Type	Count (*n* = 37/54)	Percentage (69%)
Mediastinal lymphoma	10	27%
Pulmonary metastases	10	27%
Pulmonary carcinoma	5	14%
Multicentric lymphoma	5	14%
Upper respiratory lymphoma	3	8%
Laryngeal squamous cell carcinoma	2	5%
Thyroid carcinoma	1	3%
Right auricular hemangiosarcoma	1	3%

**Table 6:** Median age of cats according to the causes of dyspnea.

Group	Count (*n* = 258)	Median Age (Years)
Respiratory	84	6
Cardiac	65	11
Trauma	55	2
Neoplasia	54	11.7

**Table 7:** Causes of pleural effusion in recent studies on cats.

Cause of Pleural Effusion	Abboud (*n* = 100)	Ruiz [21] (*n* = 380)	König [22] (*n* = 306)	Hung [23] (*n* = 220)
Cardiac origin	38%	41%	35%	53%
Pyothorax	26%	15%	9%	11%
Neoplastic origin	23%	26%	31%	20%
Traumatic origin	9%	4%	1%	6%
Feline infectious peritonitis	4%	3%	9%	1%
Chylothorax	0%	6%	5%	5%
Other causes	-	5%	10%	9%

## Data Availability

All data available on request.
